# System of Midwifery

**Published:** 1876-04

**Authors:** 


A System of Midwifery, Including Diseases of Pregnancy and the Puerperal State. By William Leishman, M.D., Regius Professor of Midwifery in the University of Glascow, Physician to University Lying-in Hospital, Corresponding Member of Obstetrical Societies of Edinburgh and Berlin, etc. Second American from the second and revised English edition; with additions by John S. Parry, M.D., Obstetrician to Philadelphia Hospital, etc. pp. 766. Philadelphia: Henry C. Lea.

It has been but a short time since the first American edition of this work was issued and noticed in this Journal; yet such is its popularity that a second has been called for, and it comes from the press not shorn of its former value, but with several new chapters on the use of the forceps, lactation, and puerperal fever. The illustrations are complete, and it is unnecessary to add words to commend it to the profession. The rapid sale and its adoption in some of the medical schools as a text-book, will be sufficient to commend it to the medical profession.

